# Paeoniflorin Improves Stroke by Modulating the ESR1 Pathway: Data Mining and Validation Based on Network Approaches

**DOI:** 10.3390/ph18070933

**Published:** 2025-06-20

**Authors:** Zhenshan Sun, Junjie Peng, Jiangbangrui Chu, Zhengyi Wang, Kefan Hu, Zhanpeng Feng, Mingfeng Zhou, Xingqin Wang, Songtao Qi, Zhu Zhang, Ken Kin Lam Yung

**Affiliations:** 1 Department of Biology, Faculty of Science, Hong Kong Baptist University, Hong Kong SAR 999077, China; 21481717@life.hkbu.edu.hk (Z.S.); hazelkkhu@gmail.com (K.H.); 2 Golden Meditech Center for Neuroregeneration Sciences, Hong Kong Baptist University, Hong Kong SAR 999077, China; 3 Teaching and Research Division, School of Chinese Medicine, Hong Kong Baptist University, Hong Kong SAR 999077, China; 22482687@life.hkbu.edu.hk (J.P.); jbrrchu@hkbu.edu.hk (J.C.); 4 Department of Neurosurgery, Nanfang Hospital, Southern Medical University, Guangzhou 510515, China; feng3388836@smu.edu.cn (Z.F.); zhoumingfengsmu@163.com (M.Z.); neurowangxq@163.com (X.W.); qisongtaonfyy@126.com (S.Q.); 5 Cuiying Honors College, Lanzhou University, Lanzhou 730000, China; wangzhengyi20@lzu.edu.cn; 6 Department of Science and Environmental Studies, The Education University of Hong Kong, Hong Kong SAR 999077, China

**Keywords:** stroke, traditional Chinese medicine, Data-Mining-Derived Approach, network pharmacology, Estrogen Receptor 1, paeoniflorin

## Abstract

***Aim of the study:*** Traditional Chinese herbs have a unique therapeutic effect on stroke and numerous successful clinical cases. However, these clinical cases are highly dispersed, creating challenges for translational research. This study employs a new paradigm to identify treatment patterns and the active compound interactions contained within these clinical cases, with experimental validation after target screening. ***Methods and Materials:*** Stroke-related targets were identified through GEO, DisGeNET, and Genecards. Active ingredients were extracted from BATMAN-TCM 2.0. All herbs and diseases were confirmed by the Pharmacopoeia of the People’s Republic of China (2020 edition) and Medical Subject Heading (MeSH). All networks in this study were constructed by Cytoscape, and data analysis was done by Python. All formulations and herbs were retrieved from the literature review. For the molecular docking process, Autodock was applied as the docking platform, and all the protein structures were downloaded from PDB. For experimental validation after target screening, HT22 cells were incubated with glucose-free DMEM and placed in an anaerobic chamber for 2 h. Subsequently, HT22 cells were reoxygenated for 24 h. Estrogen Receptor 1 (ESR1) protein levels were measured in vitro. ***Results:*** seven materials, including Angelicae Sinensis Radix, Pheretima, Chuanxiong Rhizoma, Persicae Semen, Astragali Radix, Carthami Flos, and Radix Paeoniae Rubra, were identified as the core herbs for the treatment of stroke. The targets of the stroke mechanism were screened, and the herbs-compound-target network was constructed. Among them, paeoniflorin (PF) was identified as the core active compound, and its interaction with ESR1 was verified by molecular docking as the key interaction for the treatment of stroke. In vitro experiments showed that PF inhibited cell apoptosis under hypoxia by increasing the expression of ESR1 compared with the oxygen-glucose deprivation-reperfusion (OGD/R) model group. Western showed that PF (100 μM, 200 μM) can significantly increase the decreased ESR1 protein level caused by the OGD/R model. ***Conclusions:*** seven key herbs were screened. Further bioinformatics and network pharmacology studies suggested that PF is expected to become a new active compound for the treatment of stroke. In vitro validation further demonstrated that PF enhanced neuronal survival and ESR1 expression under ischemic conditions, supporting its therapeutic candidacy.

## 1. Introduction

Stroke is an acute cerebrovascular disease that involves sudden rupture or blockage of cerebral blood vessels, leading to neuronal cell destruction and brain damage [1]. According to the Global Burden of Disease 2021 data, stroke affected 93.8 million people worldwide, with an estimated annual global cost exceeding US$890 billion, accounting for 0.66% of global GDP [2].

For the medicine development of stroke, current frontline medications offer minimal choices and can often be categorized into 3 types: antiplatelet drugs, anticoagulants, and thrombolytics. Antiplatelet drugs aim to reduce platelet aggregation, which helps with ischemic stroke but worsens the hemorrhagic stroke. The window phase of antiplatelet treatment is generally 48 h [3]. Anticoagulants like warfarin, Dabigatran, and Rivaroxaban enhance anticoagulability but elevate the risk of severe bleeding, necessitating rigorous monitoring [4]. Thrombolysis, such as the use of alteplase, is employed to dissolve blood clots and restore blood flow. This treatment necessitates administration within a 6-h window from the occurrence of stroke, with the most significant benefits observed when administered within the first three hours [5]. The constraints in the usage and types of these drugs underscore the need for ongoing research into the development of new pharmaceuticals. Therefore, new active compounds are urgently needed to treat stroke.

Traditional Chinese medicine (TCM) is a valuable repository of empirically developed traditional medicines. With the advantages of multiple targets [6] and low side effects [7], research on the treatment of stroke with TCM has made substantial progress. TCM has a traditional Chinese philosophical background and a long history, but TCM regards the human body as a complex dynamic system and usually adopts a dialectical treatment strategy to treat diseases [8]. The variety of methods for treating the same disease leads to inconsistent medical practices, and different preparation and naming methods of drugs also easily cause confusion. Coupled with the lack of evidence-based medicine verification, TCM treatment has been questioned [9]. Therefore, although TCM is effective in treating stroke, it has not yet developed into a mature medication regimen.

Bioinformatics approaches can utilize databases to identify disease-associated targets and compounds for drug development [10], with network pharmacology being one of the most well-known paradigms [11]. However, studies solely based on network pharmacology have certain limitations. One key drawback is their heavy reliance on prior knowledge, meaning that effective herbal formulas must be predefined based on existing experience. As a result, these approaches struggle to integrate diseases and molecular targets with clinical data, limiting the effective use of real-world clinical cases. Yet, many TCM prescriptions derive their strength from a long history of clinical application [12,13,14].

Therefore, to better facilitate evidence-based translation, there is an urgent need for a new paradigm that integrates clinical data mining, network pharmacology, and machine learning into a cohesive framework. This study proposes an integrated method to develop a novel Data-Mining-Driven Approach (DMDA) to improve the application of TCM in stroke treatment. The framework combines experimental validation with a cellular model to verify the priority therapeutic targets identified during computational screening. Through this multidimensional strategy, we aim to combine computational prediction with biological validation to identify the potential of TCM in stroke intervention.

## 2. Results

### 2.1. SRGs Extraction

The process of DEG selection in the volcano map is shown in Supplementary Figure S2. Then, the intersection process shows that the overlapped genes of these 3 datasets were selected. Among these 3 databases, 398 genes are screened to be the SRGs for further analysis.

### 2.2. Statistical Analysis of Formulas

**Figure 1:**
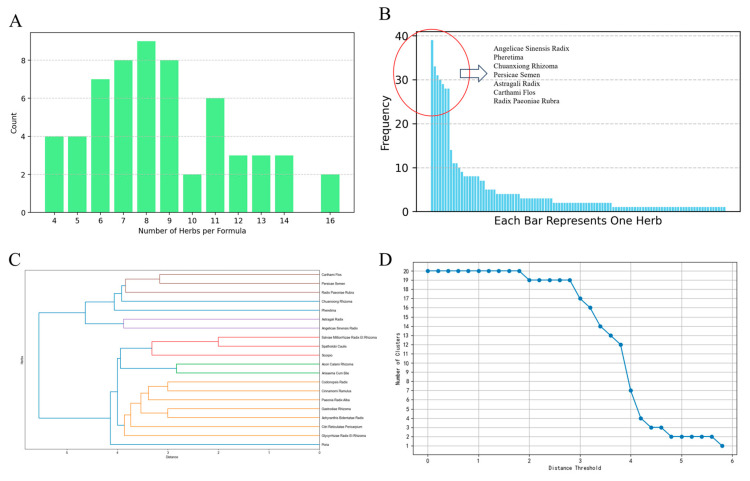
Herbal formula patterns and clustering analysis. (**A**) Distribution of the number of herbs per formula. Formulas containing 8 herbs are the most common formula. Formulas containing 8 herbs are the most common formulas for stroke treatment. (**B**) Frequency of herb usage. The usage frequency of these herbs is highly uneven. In total, the top seven herbs were used 218 times, accounting for 42.5% of the overall 513 uses, while 41 least used herbs were used only once, among the total 110 herbs. (**C**) Cluster Result of the top 20 herbs. When the distance threshold applies to 4, 5 clusters exist. Cluster 1: Angelicae Sinensis Radix and Astragali Radix; Cluster 2: Chuanxiong Rhizoma, Persicae Semen, Radix Paeoniae Rubra and Carthami Flos; Cluster 3: Poria; Cluster 4: Pheretima; Cluster 5: rest of the 12 herbs. (**D**) Number of significant decrease in the number of clusters when the threshold is set to 4. At this elbow point, the slope of the curve is the steepest, indicating the ideal threshold point.

The distribution of the number of herbs per formula is shown in Figure 1A. The frequency of herb usage shows that seven herbs have a relatively higher frequency. The significant difference in frequency between the top seven herbs and other herbs can be observed in Figure 1B, which shows the overall frequency distribution of different herbs. In hierarchical clustering, the detailed clustering process and the selection of the distance threshold are shown in Figure 1C,D.

**Figure 2:**
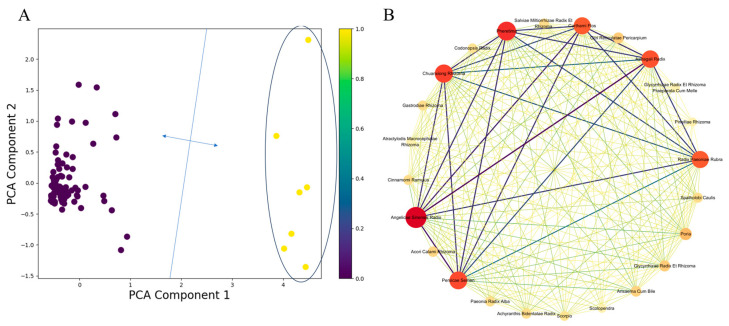
Clustering and association analysis of herbal formulas. (**A**) The K modes after PCA. The boundaries between the 2 clusters appear extremely distant, indicating clear classification. Notably, the rightward cluster (in the deep blue circles) contains seven different types of herbs, which are exactly those previously mentioned herbs that have significant enrichment in usage. This, to some extent, reveals the regularity of their formulas: they heavily utilize these herbs. (**B**) Apriori association network. Herbs with higher frequency are expressed using bigger sizes. A deeper herb node color indicates a greater number of associations with other herbs. The thickness of the network connections represents the lift. The edge lines vary from deep purple to light yellow, with deeper colors corresponding to higher confidence.

For K mode clustering, the clustering performance is best when the number of clusters applies 2, at which point the distance between these 2 clusters is wide and distinct. After clustering, we performed PCA to reduce the dimension of the cluster result for proper visualization. Figure 2A shows the K modes clustering result after PCA. To further explore the formulas’ regularity and to identify the core herbs, we conducted an Apriori association analysis. Under the threshold of support ≥ 15%, confidence ≥ 80%, lift ≥ 1.2, there were 52 association rules remaining, including 2 two-itemset association rules and 50 three-itemset association rules. The detailed association rules are shown in Supplementary Figure S7.

After calculating the association of the common herbs, a network graph (Figure 2B) was introduced to visualize the association analysis result. In this network, we can see seven core nodes, namely Angelicae Sinensis Radix, Pheretima, Chuanxiong Rhizoma, Persicae Semen, Astragali Radix, Carthami Flos, and Radix Paeoniae Rubra. This indicates that these seven herbs not only have the highest usage frequency but also interact closely with other herbs. Moreover, the interactions among the seven herbs are also tight, forming an absolute core.

### 2.3. Compound and Target Screening Result

The compounds and corresponding targets of these seven herbs were retrieved. All the amounts of active compounds and targets for each drug are shown in Table 1. The “Multi-Origin” column stands for the active compounds with multiple origins. After overlapping the SRGs and the targets that these seven herbs can affect, a number of 192 stroke-related targets for these seven herbs were identified. Table 1 summarizes the active compound amounts and the corresponding target amounts for these active compounds in each kind of herb.

### 2.4. Results of PPI Analysis for Core Target Selection

After removing the isolated, single nodes, a PPI network was established. Supplementary Figure S6 shows the overall structure of the PPI network, with the top herb targets highlighted in the center.

### 2.5. Drug-Compound-Target Analysis Result

**Figure 3:**
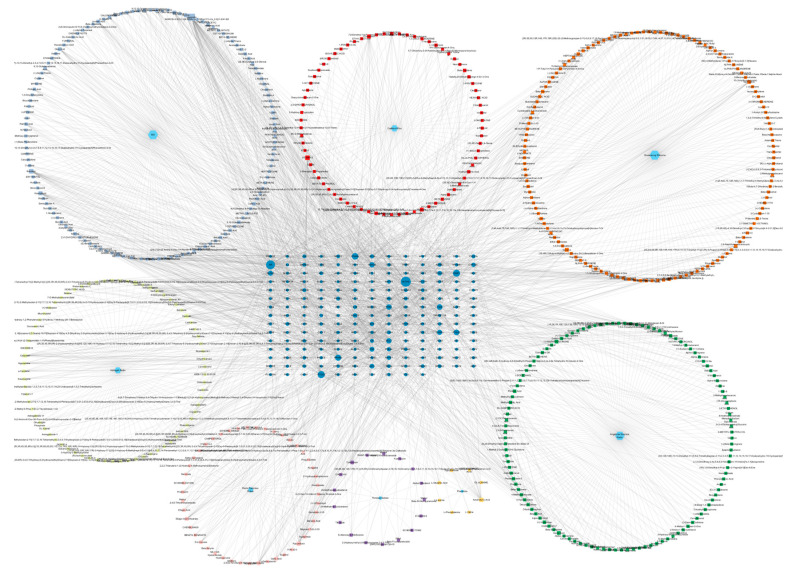
Comprehensive herb-compound-target network.

We built the drug-compound-target network based on the multi-components and multi-target features in these seven TCM herbs, shown in Figure 3. This network possesses 803 nodes and 2542 edges. Such a network with high density effectively displays the potency of each herb, the most efficacious components within one herb, and the disease target with the highest centrality. The top 10 active compounds shown in the network are quercetin, baicalein, baicalin, vitamin E, daidzein, arachidonic acid, PF, kaempferol, capsaicin, and calycosin. Among them, quercetin, baicalein and PF were selected for further study.

The network displays the comprehensive scale of the drug-compound-target interaction. Each medicinal material is represented in a light blue hexagon; the square “MO” stands for “multi-origin”, referring to the compounds that have multiple origins from the seven herbs. The circles of dark blue stand for the targets. Active compounds are marked as different colors of squares in the network. Among these compounds, the top three with the highest number of targets were quercetin (74 targets), baicalein (31 targets), and paeoniflorin (24 targets).

### 2.6. Key Molecular Selection and Molecular Docking Results

The key targets of these seven herbs were identified as the intersection of the top 20 SRGs and the top 20 core targets. Through the overlapping process, seven targets, including TNF, AKT1, CASP3, BCL2, PTGS2, PPARG, and ESR1, were selected, and 4 of them were selected for further molecular docking verification. Table 2 shows the detailed active compounds, target gene symbol, and PDB ID of the selected protein structure file.

**Figure 4:**
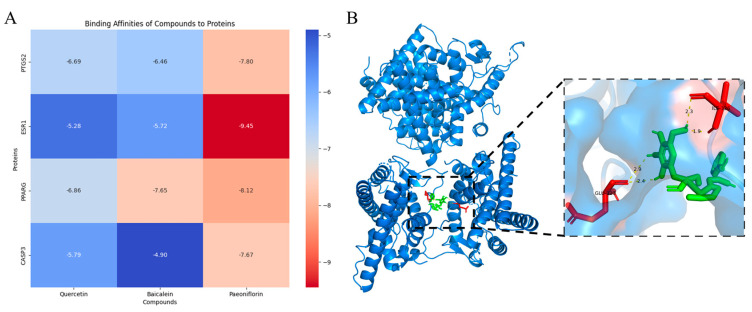
Molecular docking analysis of active compounds. (**A**) The heatmap shows the binding energy of each molecular docking. (**B**) The molecular docking result of PF and ESR1 has the lowest binding energy. PF is recognized in green structures. The docking result suggests that 4 Hydrogen bondings were formed with different lengths. The hydrogen bondings are marked in bright yellow. The amino acid residues that are connected to the hydrogen bonding are marked in red.

After molecular docking, the binding energy of each pair of targets and active compounds combination was collected and visualized in a heatmap (Figure 4A).

Most of the target-compound docking results demonstrate a high degree of stability, with binding energies below −5 kcal/mol. The most potent interaction was observed between PF and ESR1, which showed a binding energy of −9.45 kcal/mol. This indicates a highly promising binding affinity, suggesting great potential for drug development. The detailed docking simulation of PF and ESR1 is shown in Figure 4B.

The molecular docking result further shows that the core molecular mechanism of these seven herbs for stroke treatment shall be the interaction of PF and ESR1. While other compounds-targets interaction is valuable as well, the aforementioned interaction has the most potential for drug development.

### 2.7. PF Increased the Viability and Increased ESR1 Expression Level of OGD/R Model Cells

**Figure 5:**
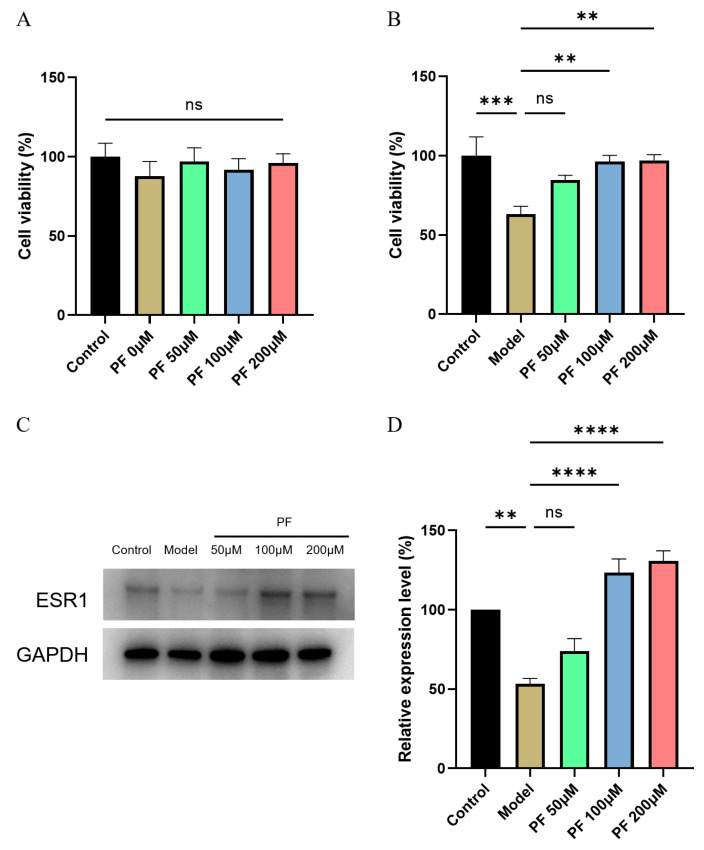
Validation of PF-ESR1 interaction on cell models. (**A**) MTT measurement results after PF treated for 24 h. n = 6. (**B**) Cell morphology of control, model, and model  +  PF groups. Results are expressed as mean  ± SEM. n = 12. *** *p*  <  0.001 compared with the control group, ** *p*  <  0.01 compared with the model group. (**C**,**D**) Relative expression of ESR1 of control, model, and model  +  PF groups. Results are expressed as mean ± SEM. n = 3. *** *p*  <  0.001 compared with the control group, **** *p*  <  0.0001 compared with the model group.

To gain insight into the pharmacological effects of PF in OGD/R model cells, we first investigated the effects of several doses of PF (0, 50, 100, 200 μM) on HT22 for 24 h, and no significant differences were found, indicating that PF treatment was safe (Figure 5A). OGD/R injury significantly reduced the viability of HT22 cells (*p*  < 0.001), and PF (100 μM and 200 μM) increased the viability of HT22 cells (*p*  < 0.01, Figure 5B). To prove whether PF works through ESR1, the results of Western showed that OGD/R injury significantly reduced the expression of ESR1 in HT22 cells (*p*  < 0.001). PF treatment increased ESR1 protein expression compared with the OGD/R model group (*p*  < 0.0001, Figure 5C,D). These findings suggest that PF itself does not affect the cellular state of HT22 cells and can protect cell viability under oxygen-glucose deprivation conditions.

## 3. Discussion

Though there is some research on TCM treatment for stroke [15], there are no systematic data-mining studies based on multiple clinical projects; our research filled the blank. In our study, we used a variety of statistical methods and several ML algorithms to perform a DMDA holistic analysis to discover the regularity of TCM herb usage for stroke treatment. In this DMDA, we used data from hundreds of clinically effective cases, which greatly enhanced the utility and confidence level of this study. This further demonstrates the unique strength of DMDA: using massive data to overcome the randomness or bias of individual experiments.

Our DMDA approach offers significant advantages over traditional network analysis. While network pharmacology can serve as a useful starting point for identifying potential active compounds, it remains a highly predictive framework and, fundamentally, speculative in nature. A major limitation of conventional network pharmacology is that it tends to yield results regardless of input quality. This often encourages over-interpretation or selective reporting of findings that merely align with preconceived expectations, thereby resulting in a high risk of false positives. In contrast, our DMDA approach is grounded in clinically informed translational research. The “seven herbs” were identified through extensive analysis of positive clinical case-control studies. By integrating data mining, algorithmic screening, network pharmacology, and experimental validation, DMDA provides a robust framework for translating clinical experience into actionable insights for drug development.

For instance, many studies face a significant limitation by relying solely on a single database [16,17], while other studies on aging diseases lack demographic baseline control [18]. On the other hand, our study integrated multiple databases and ensured a balanced demographic baseline, which helped to overcome most statistical biases. This approach allowed us to identify high-confidence disease targets, laying a solid foundation for subsequent research. In the analysis of SRGs, the hub genes highlighted the key modules within SRGs, and the significance of these genes/targets has already been noted in previous studies. Additionally, we provided several clinical insights for drug development. For example, previous research pointed out that AKT1 does not affect stroke and augmentation of this pathway may not be a feasible approach to neuroprotection in stroke [19]; later research then came out with opposite ideas: the role of AKT1 in stroke neuroprotection is important, and active compounds such as H2S/Atorvastatin can attenuate the damage through pathways related to AKT1 [20,21]. Now, according to our DMDA approaches, with the filtering effect from the big data, we can settle this controversy at a high confidence level: the significance of AKT1 in the pathogenesis of stroke is undeniable.

Beyond PF, our network pharmacology analysis identified several other bioactive compounds with potential therapeutic relevance for stroke, including quercetin, baicalein, and calycosin (Figure 3). These compounds exhibited strong binding affinities to key stroke-related targets such as CASP3 and PPARG (Figure 4A), suggesting their possible pharmacological activity. For instance, quercetin and baicalein have demonstrated neuroprotective effects in previous studies through anti-inflammatory and antioxidant pathways, which may complement PF’s ESR1-mediated mechanism.

It is well known that one of the key advantages of TCM lies in its multi-target effects and inherent synergistic actions [22]. This feature is also reflected in our study. As shown in Figure 3, among all the compound subnetworks within the herb–compound–target network, the most critical module is composed of MO (multi-origin) compounds. This indicates that the seven botanical drugs discussed in this study exhibit inter-herb synergy by jointly contributing shared compounds that regulate the disease network. This finding is consistent with previous studies, such as the one by Zhang et al. (2025) [23], which have similarly highlighted synergistic patterns in TCM formulations.

Furthermore, beyond the ESR1–PF interaction, our analysis also identified multiple potential PF–target interactions, including the PTGS2, PPARG and CASP3, and these targets may synergistically promote neuroprotection with ESR1 in the action of PF. Due to limited experimental resources, we were unable to validate all of them individually. However, their presence underscores the characteristic of TCM in treating stroke through multi-compound, multi-target synergistic mechanisms. While our current study focused on validating the dominant PF-ESR1 interaction, future research should systematically evaluate potential synergies among these compounds using combination index assays and multi-target activity profiling.

Regarding the experimental design, we acknowledge that the inclusion of positive control (e.g., clinical neuroprotectants) and ESR1 blocker/antagonists/gene knockdown would strengthen the cellular validation. This limitation was due to technical constraints in matching the pharmacological profiles of existing stroke drugs with our specific OGD/R-ESR1 mechanism. However, the consistency between our computational predictions (−9.45 kcal/mol binding energy for PF-ESR1) and experimental results (dose-dependent ESR1 upregulation) provides internal validation of our findings. Future studies will incorporate appropriate positive controls such as estradiol or selective ESR modulators to enable direct efficacy comparisons. Also, the neuroprotective effect of PF on stroke needs to be verified in in vivo animal models, such as the middle cerebral artery occlusion (MACO) model in mice, to confirm its translational relevance.

TCM has a wealth of clinical practice experience, but this knowledge often cannot be directly translated into drug development insights. This limitation is primarily due to the non-standardized approach of TCM syndrome differentiation and treatment [24], as well as traditional methodologies [25], which result in a significant amount of redundancy and noise within the clinical data. Different TCM pharmacists, due to their personal experience and clinical style, have variations in assigning prescriptions [26]. For instance, in our study, there are as many as 56 different oral formulations for stroke in the last 10 years. However, through our DMDA, a common pattern among these formulations has emerged: the consistent use of these seven herbs. Frequency analysis revealed a significant enrichment of these seven herbs, while clustering analysis distinctly separated them from other herbs. Association analysis further highlighted their central role within the formulation network and demonstrated their interconnections with each other. These seven core herbs may contribute to the extraction of active compounds and the development of new drugs in the future.

Furthermore, our DMDA leveraged network pharmacology and computational methods to illustrate the active target-compound interaction. Molecular docking shows that PF generally exhibits stronger binding affinity to several key targets compared to other candidate compounds, which distinguishes it as a potentially druggable compound for stroke. Our in vitro OGD/R model further validated this interaction: PF treatment significantly restored HT22 cell viability and reversed OGD/R-induced ESR1 downregulation, aligning with the molecular docking prediction of PF-ESR1 binding energy. These findings suggest that PF exerts neuroprotective effects by enhancing ESR1 expression, a mechanism consistent with prior studies linking ESR1 activation to ischemic stroke recovery through anti-apoptotic and anti-inflammatory pathways [27,28]. Notably, PF alone did not alter baseline cell viability, underscoring its safety profile in non-ischemic conditions. Extracting raw data from clinical treatment case reports, we gradually focused on several active compounds through this DMDA approach. Simultaneously, bioinformatics data from clinical samples allowed us to identify the target sites of these active ingredients. By combining these two sources, we established an efficient research paradigm for translational studies, which effectively provides novel insights for drug development. The synergy between computational predictions and experimental validation—particularly the PF-ESR1 axis—highlights the robustness of our DMDA framework. This approach bridges the gap between TCM clinical data and mechanistic studies, addressing the skepticism surrounding TCM’s evidence base [9]. The abundance of clinical data lends high confidence to the ESR1-PF interaction, suggesting that subsequent drug development based on this interaction holds significant promise. This DMDA paradigm can be applied not only to TCM for stroke treatment but also to other ethnopharmacological studies, offering valuable insights for the modernization and translational development of traditional medicines. Paradigm can be applied not only to TCM for stroke treatment but also to other ethnopharmacological studies, offering valuable insights for the modernization and translational development of traditional medicines.

## 4. Materials and Methods

### 4.1. Reagents

Dulbecco’s modified Eagle medium (DMEM), fetal bovine serum (FBS), penicillin/streptomycin (P/S), and trypsin-EDTA were obtained from Gibco (Grand Island, NY, USA). Dimethyl sulfoxide (DMSO), 3-[4,5-dimethylthiazol2-yl]-2,5-diphenyltetrazolium bromide (MTT), paeoniflorin (PF, obtained from Chengdu Must Bio-technology Co., Ltd., Chengdu, China), bicinchoninic acid (BCA) assay kit.

### 4.2. Establishing the Searching Strategy

**Figure 6:**
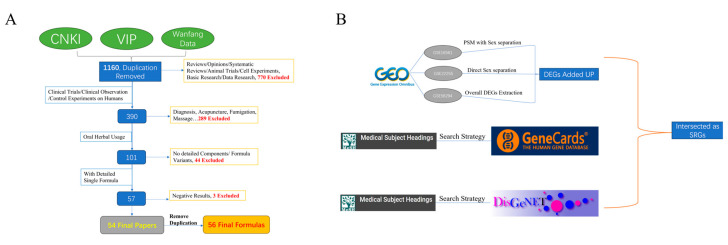
Selection criteria and process formulas and stroke-related genes (SRGs). (**A**) Detailed selection criteria for formulas. (**B**) The overall selection process of SRGs.

The standardized terms for stroke are defined by Medical Subject Heading (MeSH); specific search terms are detailed in Supplementary Figure S1. Meanwhile, in GEO, 3 datasets, GSE16561, GSE22255, and GSE58294, are selected, including 128 stroke patients and 67 healthy controls are obtained. As for the stroke-treating formulas, we conducted a review of clinical studies on TCM treatment for stroke over the past ten years from the CNKI, VIP, and Wanfang knowledge base. The selection criteria are shown in Figure 6A. After going through these selections, a total number of 54 papers were selected, with 56 different formulas. The *Pharmacopoeia of the People’s Republic of China 2020 Edition* was used to standardize the Latin names of these herbs.

### 4.3. Extraction of SRGs

Vital covariates of stroke occurrence and progression include age and sex [29]. Therefore, when comparing patients with healthy controls to expose differentially expressed genes (DEGs, Supplementary Figure S2), aside from dataset GSE58294, males and females in the other two datasets were analyzed separately. DEGs between patients and control groups were identified for each sex first and then the union afterward. As for GSE58294, which contains 69 stroke patients and 23 healthy controls, the original publisher stated that there was no statistical significance in age, sex, and other baseline factors [30]. Therefore, the DEGs were directly extracted from the overall comparison between patients and controls. For GSE22255, which contains 20 patients and 20 healthy controls, the Mann-Whitney U test for age distribution yields a *p*-value of 0.43, and the Chi-squared test for sex distribution yields a *p*-value of 1, indicating there is no significant difference between the 2 groups in age and sex distribution. For GSE16561, which contains 39 patients and 24 healthy controls, the initial distribution of age and sex is uneven between the control group and the patient group (PSM) to balance the baseline characteristics between the two sample groups. After the PSM, the Mann-Whitney U test for age distribution yields a *p*-value of 0.11, and the Chi-squared test for sex distribution yields a *p*-value of 1, indicating that the age and sex are well-balanced after PSM. Supplementary Figure S3 shows the age and sex distribution of GSE16561 after PSM and GSE22255. For DisGeNET and GeneCards, SRGs were directly acquired using the previous search strategy. The final SRGs were identified as the intersection of these 3 bunches of genes from GEO data, DisGeNET, and GeneCards. The overall selection process is visualized in Figure 6B.

### 4.4. Formulas Pattern Analysis

Using Python 3.10, we analyzed the appearance frequency of each herb and the distribution of the number of herbs in different herbal formulas. The herbs with the top 20 frequency were selected for cluster analysis. Two Machine Learning (ML) clustering algorithms, hierarchical clustering and K-mode clustering, were utilized to classify different usage features of these herbs. To reveal the herbal co-occurrence regularities, the Apriori algorithm was used for association analysis on these herbs. Core herbs were screened for further analysis.

### 4.5. Active Compounds and Corresponding Target Screening for 7 Herbs

We retrieved the active compound and the related protein target of these core herbs based on the BATMAN-TCM 2.0 database [31]. Both the known targets and the predicted potential targets with a score ≥ 0.84 were added up as the target corresponding to the active compound in the herb.

### 4.6. Stroke-Related Targets Analysis

By intersecting the SRGs and the targets corresponding to the active compounds, we identified specific genes/proteins that may be affected by these herbs in the context of stroke. These stroke-related targets allow for a focused analysis of how these herbal compounds could potentially affect the biological mechanism and pathway of stroke, providing biomedical insights. To understand the biological background of these stroke-related targets, Gene Ontology (GO) [32] and Kyoto Encyclopedia of Genes and Genomes (KEGG) [33] pathway functional enrichments were conducted. Protein–Protein Interaction (PPI) analysis was performed based on STRING [34] (Supplementary Figures S4 and S5). Supplementary Figure S6 shows the overall structure of the PPI network, with the top herb targets highlighted in the center.

### 4.7. Construction of Drug-Compound-Target Network

To illustrate the comprehensive, multi-target effect of these 7 herbs in stroke treatment, a network analysis was performed. The targets and compounds were inputted into the Cytoscape (version 3.10.2) [35] together with the TCM drug origin to construct the “drug-compound-target” network.

### 4.8. Key Molecular Interaction Identification with Molecular Docking Verification

The top 20 nodes with the highest degree from the SRGs’ PPI network and the top 20 nodes with the highest degree from the stroke-related targets PPI network were selected and overlapped. The intersection of these 2 groups of genes/proteins was identified as the core targets affected by these 7 herbs. Then, a series of molecular docking was conducted to verify the interaction between the 3 selected core compounds and 4 selected core targets.

### 4.9. Cell Line and Cell Culture

HT22 cell was purchased from ATCC (Rockville, MD, USA). HT22 cells were cultured in DMEM supplemented with 10% FBS and 100 U/mL P/S. The cells were cultured at 37 °C under 5% CO_2_, and the medium was changed every 2–3 days.

### 4.10. Cell Viability Assay

To investigate the effects of PF on the cell growth in HT22 cells, cell viability was measured by MTT assay. HT22 (1 × 10^4^ cells/well) were seeded in a 96-well plate and then incubated for 24 h. The cells were treated with various concentrations of PF (0, 50, 100, 200 μM). After the indicated time, 10 μL of MTT (5 mg/mL) was added to each well, and then formazan crystals were dissolved in 100 μL of DMSO. The absorbance was measured at 570 nm using a spectrophotometer.

### 4.11. Experimental Cell Groups and Oxygen-Glucose Deprivation-Reperfusion (OGD/R) Model

The cells were divided into 5 groups: control group, OGD/R model group, and OGD/R model + PF group (PF 50 μM, 100 μM, 200 μM). The OGD/R model cells were cultured with glucose-free DMEM, and the OGD/R model + PF were cultured with PF-containing glucose-free DMEM. The glucose-free DMEM was pretreated within an anaerobic chamber (37 °C, 94% N_2_, 5% CO_2_) overnight and allowed to equilibrate with the hypoxic atmosphere. Then both groups were placed in an anaerobic chamber (37 °C, 94% N2, 5% CO_2_) for 2 h [36,37]. Subsequently, cells were reoxygenated in a 37 °C, 5% CO_2_ incubator for 24 h. At the beginning of reoxygenation, the OGD/R group was placed in DMEM for 24 h, and the OGD/R + PF group was placed in a PF-containing DMEM for 24 h.

### 4.12. Western Blotting

To prepare the sample, HT22 cells were lysed on ice for 30 min with RIPA lysis buffer containing protease inhibitors, the supernatant was centrifuged, and the protein concentration was determined by BCA method, and then mixed with 4× SDS loading buffer and boiled for 5 min. SDS-PAGE electrophoresis was then performed using a 10% separation gel and a 5% concentrated gel system, and the sample was loaded on each well of the concentrated gel, and then the protein was transferred to a PVDF membrane. After transfer, the membrane was blocked with 5% skim milk/TBST for 1 h, and diluted primary antibodies (GAPDH 1:2000; ESR1 1:1000) were added in sequence and incubated overnight at 4 °C and HRP-labeled secondary antibodies (Rabbit, 1:5000) were incubated at room temperature for 1 h, and finally developed with ECL chemiluminescent reagents, and the signal was collected by an imaging system. Independent experiments were repeated 3 times. Image J was used to compare the density of bands.

### 4.13. Statistics Analysis

All data are presented as mean ± SEM. Statistical analysis of three or more groups was performed by one-way ANOVA and Dunnett’s multiple comparisons using GraphPad Prism version 10.0 software (USA). Values of *p*  <  0.05 were considered statistically significant. The selection of DEGs in the volcano plot was based on a stringent threshold of p_adj_ < 0.01. For GeneCards, genes with association scores above the median were extracted. For DisGeNET, only genes with a gene-disease association score greater than 0.05 were considered. For PPI analysis, to increase the confidence of the interactions among SRGs, we obtained the interaction with a total score ≥ 0.4 and excluded the relation based on text-mining, databases, and gene-neighborhood.

## 5. Conclusions

In summary, this research extracted the key regularities of TCM stroke treatment. Further bioinformatics and network pharmacology studies suggest that the interaction between ESR1 and PF shows promise in the development of new active compounds for stroke. Experimental validation in OGD/R models confirmed that PF significantly restored neuronal viability and upregulated ESR1 expression, reinforcing its therapeutic potential. The DMDA in our research can aid the translational studies for clinical and drug development of ethnopharmacology as a paradigm.

## Figures and Tables

**Table 1:** Active Compounds and Target Counts for Each Herb.

Herb	Compound Amount	Corresponding Target Amount
Chuanxiong Rhizoma	202	506
Angelicae Sinensis Radix	153	408
Pheretima	11	236
Radix Paeoniae Rubra	74	419
Carthami Flos	121	476
Astragali Radix	87	478
Persicae Semen	21	137
Multi-Origin	170	884

**Table 2:** Active Compounds, Target Genes, and PDB IDs for Molecular Docking.

Gene Symbol	PDB ID	Active Compound
CASP3	1RHJ	Quercetin Baicalein Paeoniflorin
PPARG	8WFE	
PTGS2	5F19	
ESR1	7UJO	

## Data Availability

The underlying code for this study is not publicly available but may be made available to qualified researchers at reasonable request from the corresponding author. Yung Ken Kin Lam (kklyung@eduhk.hk) will handle the requests.

## Supplementary Materials

The following supporting information can be downloaded at: https://www.mdpi.com/article/10.3390/ph18070933/s1.

## Abbreviations

BCA, bicinchoninic acid; DEGs, differentially expressed genes; DMDA, Data-Mining-Derived Approach; DMEM, Dulbecco’s modified Eagle medium; DMSO, Dimethyl sulfoxide; ESR1, Estrogen Receptor 1; FBS, fetal bovine serum; GO, Gene Ontology; KEGG, Kyoto Encyclopedia of Genes and Genomes; MeSH, Medical Subject Heading; ML, Machine Learning; MTT, 3-[4,5-dimethylthiazol2-yl]-2,5-diphenyltetrazolium bromide; OGD/R, oxygen-glucose deprivation-reperfusion; PF, paeoniflorin; PPI, Protein-Protein Interaction; P/S, penicillin/streptomycin; PSM, propensity score matching; SRGs, stroke-related genes; TCM, traditional Chinese medicine; PCA, principal component analysis.
