# Supplementary material for: Paeoniflorin Improves Stroke by Modulating the ESR1 Pathway: Data Mining and Validation Based on Network Approaches
Source: Pharmaceuticals (Basel). 2025 Jun 20;18(7):933. doi: 10.3390/ph18070933 (PMC12299786; doi:10.3390/ph18070933)
Supplement: Supplementary file 1 [file pharmaceuticals-18-00933-s001.zip › pharmaceuticals-3609146-supplementary.pdf]

Supplements

Database	Search Term
**GEO**	("Stroke" OR "Brain Infarction" OR "Hemorrhagic Stroke" OR "Ischemic Stroke" OR "Brain Stem Infarction" OR "Cerebral Infarction" OR "Embolic Stroke" OR "Thrombotic Stroke")
**GeneCards**	("Brain Infarction" OR "Brain Stem Infarction" OR "Cerebral Infarction" OR "Embolic Stroke" OR "Hemorrhagic Stroke" OR "Ischemic Stroke" OR "Stroke" OR "Thrombotic Stroke")
**DisGeNET**	("Brain Infarction" OR "Cardioembolic Stroke" OR "Cerebral Infarction" OR "Cerebrovascular Accident" OR "Embolic Stroke" OR "Hemorrhage Stroke" OR "Ischemic Stroke" OR "Lacunar Stroke" OR "Middle Cerebral Artery Infarction" OR "Thromboembolic Stroke" OR "Thrombotic Stroke")

Sup Figure S1. The search strategy in each database.

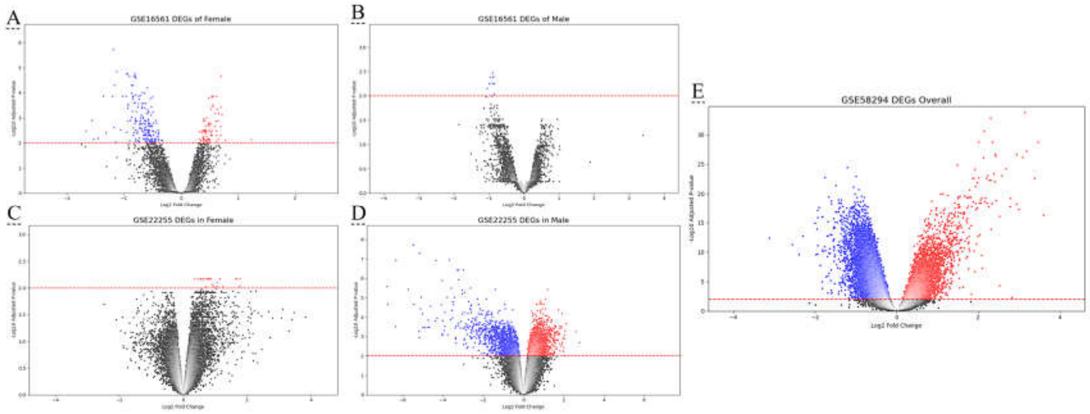

Sup Figure S2. is the volcano map for DEGs selection. A strict threshold of $p_{adj}<0.01$ was applied as the red line shown. Figures A&B show the DEGs for females and males in GSE16561, respectively. Figure C&D is the DEGs for females and males in GSE22255 respectively. Figure E is the overall DEGs revealed in GSE58294. The DEGs are all added up for later cross-database selection.

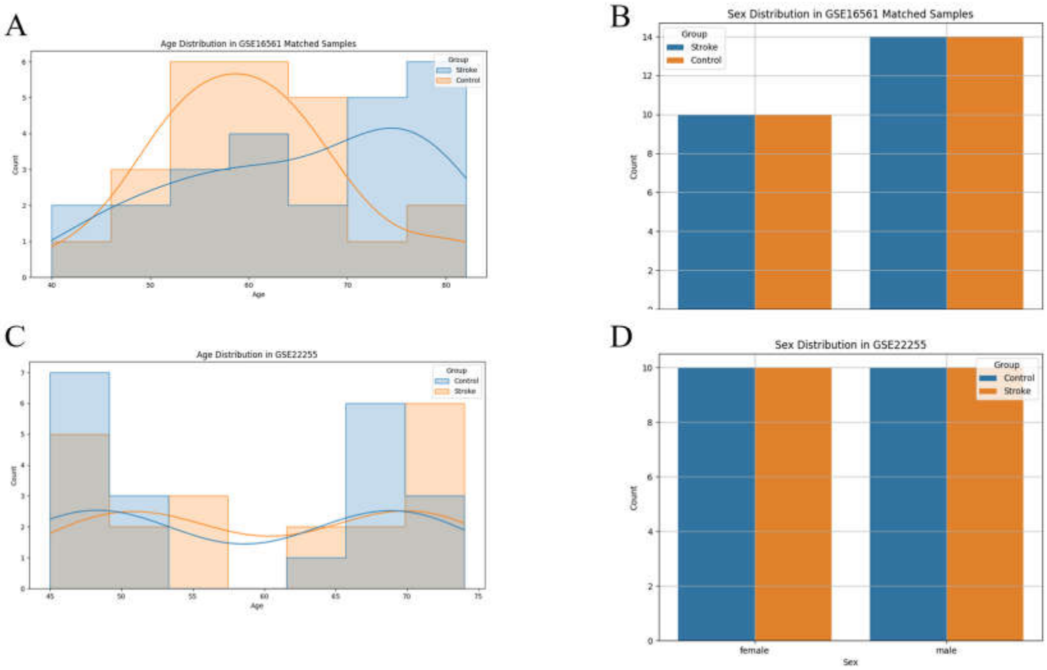

Sup Figure S3. A-D are the age distribution in GSE16561 after PSM, the sex distribution in GSE16561 after PSM, the age distribution in GSE22255, and the sex distribution in GSE22255, respectively. In both datasets, sex distribution is even, and the age distribution between the control group and the patient group is balanced.

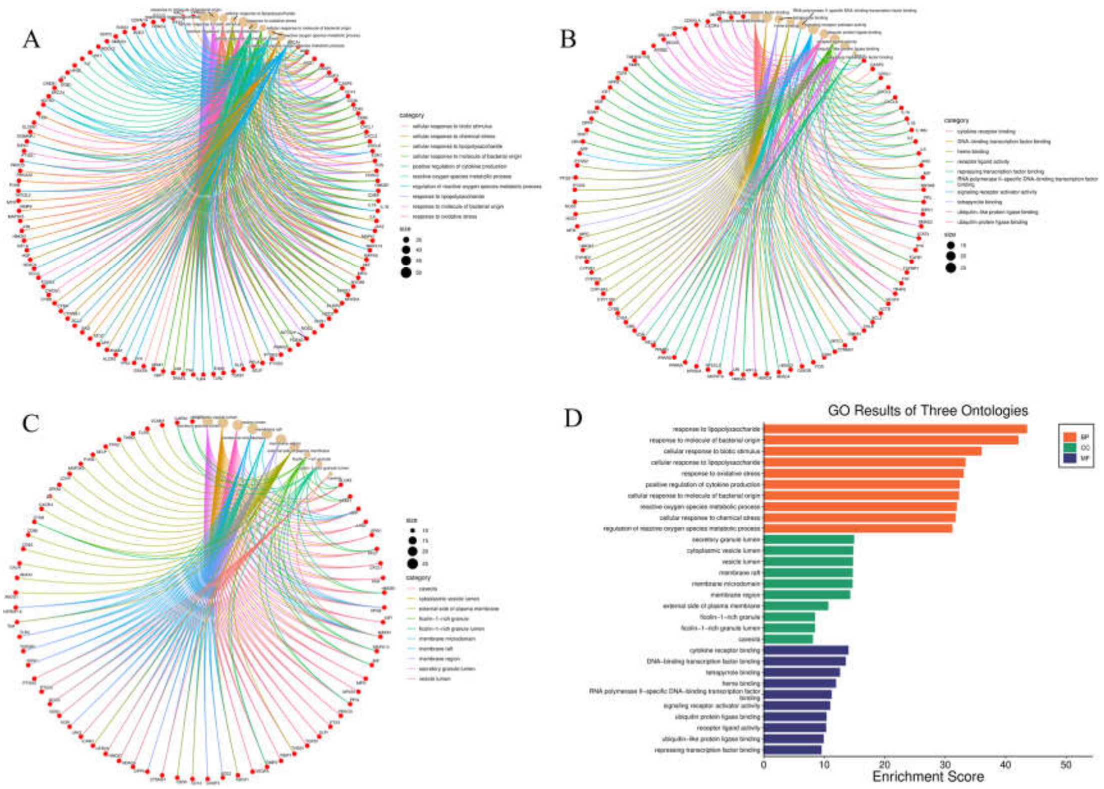

Sup Figure S4. subplot A, subplot B, subplot C show the CNet enrichment results of BP, CC and MF respectively. In the CNet plots, the genes within each enrichment term are plotted as red dots, while the enrichment sets are drawn in light brown. The size of the terms represents the gene amounts that this set contains in this enrichment. The bar chart in subplot D reflects the overall significance of the enrichment result. Enrichment Score = -$\log_{10}$(p-value).

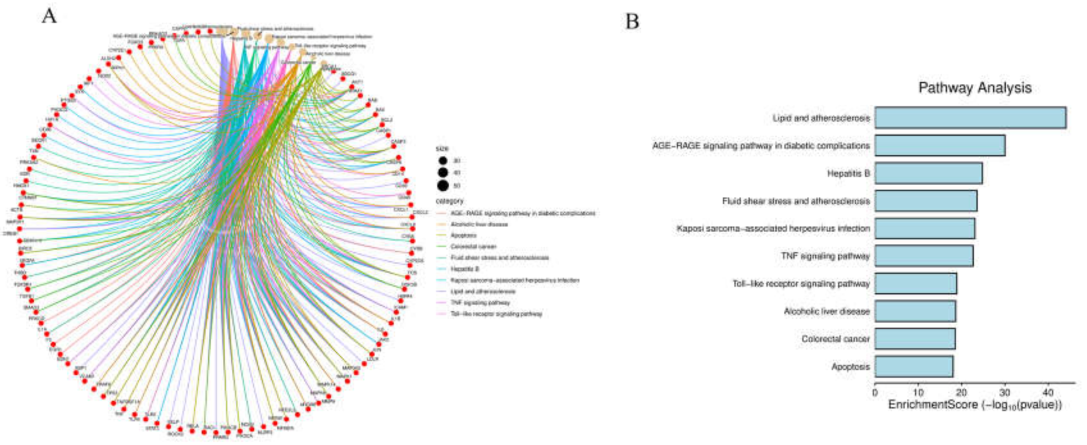

Sup Figure S5. A shows the detailed top 10 enriched pathways while subplot B shows the significance of these pathway enrichments.

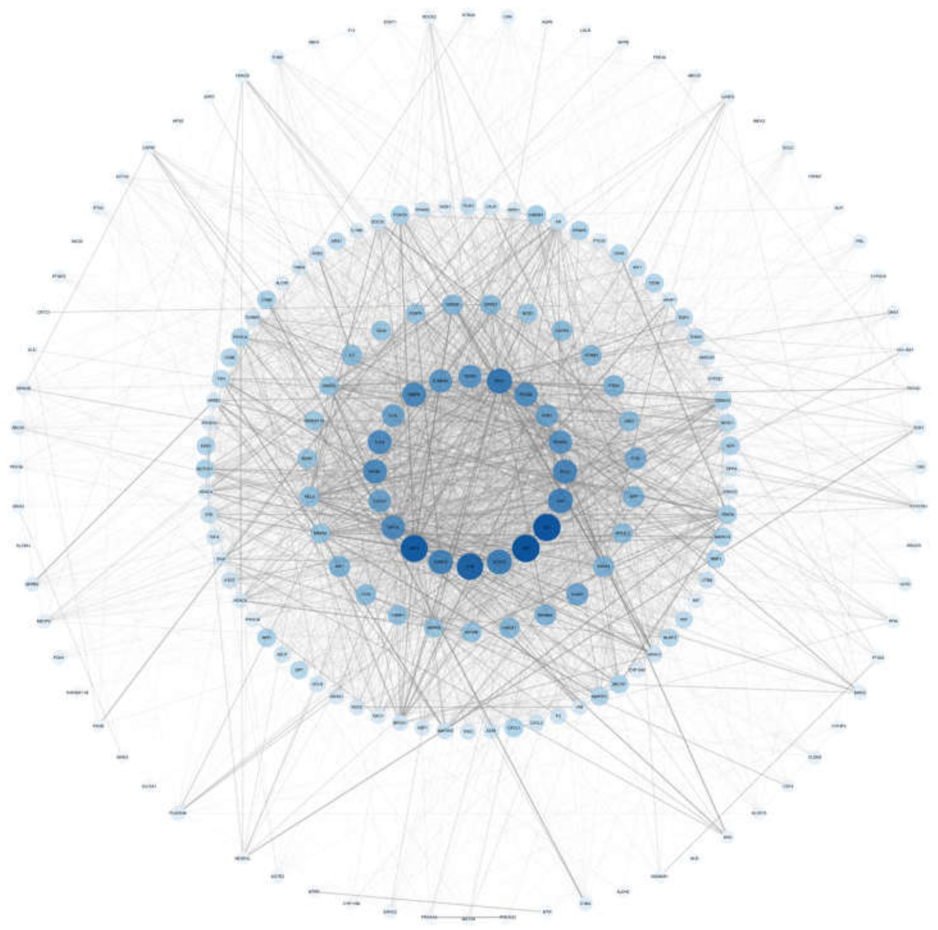

Sup Figure S6. is the PPI network for top herb target identification. The boldness of the gray line stands for the strength of the interaction. Gradient size and transparency are used to highlight the centrality degree of each node. The top 20 core targets are placed in the core of this map while relatively marginal genes are placed in the outside layers

Antecedent	Consequent	Support	Confidence	Lift
**'Carthami Flos'**	'Persicae Semen'	0.38	0.84	1.62
**'Astragali Radix'**	'Angelicae Sinensis Radix'	0.43	0.89	1.38
**'Carthami Flos', 'Chuanxiong Rhizoma'**	'Persicae Semen'	0.34	1	1.93
**'Carthami Flos', 'Persicae Semen'**	'Chuanxiong Rhizoma'	0.34	0.9	1.69
**'Persicae Semen', 'Chuanxiong Rhizoma'**	'Carthami Flos'	0.34	0.9	2.03
**'Carthami Flos', 'Chuanxiong Rhizoma'**	'Angelicae Sinensis Radix'	0.29	0.84	1.31
**'Radix Paeoniae Rubra', 'Carthami Flos'**	'Persicae Semen'	0.29	0.84	1.63
**'Radix Paeoniae Rubra', 'Persicae Semen'**	'Carthami Flos'	0.29	0.94	2.11
**'Radix Paeoniae Rubra', 'Persicae Semen'**	'Angelicae Sinensis Radix'	0.25	0.82	1.28
**'Carthami Flos', 'Angelicae Sinensis Radix'**	'Persicae Semen'	0.32	0.9	1.74
**'Carthami Flos', 'Persicae Semen'**	'Angelicae Sinensis Radix'	0.32	0.86	1.33
**'Angelicae Sinensis Radix', 'Chuanxiong Rhizoma'**	'Persicae Semen'	0.32	0.86	1.66
**'Persicae Semen', 'Chuanxiong Rhizoma'**	'Angelicae Sinensis Radix'	0.32	0.86	1.33
**'Radix Paeoniae Rubra',**	'Persicae Semen'	0.29	0.84	1.63

**'Chuanxiong Rhizoma'**				
**'Radix Paeoniae Rubra', 'Persicae Semen'**	'Chuanxiong Rhizoma'	0.29	0.94	1.76
**'Persicae Semen', 'Astragali Radix'**	'Angelicae Sinensis Radix'	0.34	0.95	1.48
**'Persicae Semen', 'Angelicae Sinensis Radix'**	'Astragali Radix'	0.34	0.83	1.71
**'Carthami Flos', 'Astragali Radix'**	'Persicae Semen'	0.3	0.85	1.64
**'Carthami Flos', 'Persicae Semen'**	'Astragali Radix'	0.3	0.81	1.68
**'Persicae Semen', 'Astragali Radix'**	'Carthami Flos'	0.3	0.85	1.9
**'Astragali Radix', 'Chuanxiong Rhizoma'**	'Persicae Semen'	0.3	0.94	1.82
**'Persicae Semen', 'Astragali Radix'**	'Chuanxiong Rhizoma'	0.3	0.85	1.59
**'Persicae Semen', 'Chuanxiong Rhizoma'**	'Astragali Radix'	0.3	0.81	1.68
**'Radix Paeoniae Rubra', 'Angelicae Sinensis Radix'**	'Astragali Radix'	0.29	0.84	1.75
**'Radix Paeoniae Rubra', 'Astragali Radix'**	'Angelicae Sinensis Radix'	0.29	0.84	1.31
**'Radix Paeoniae Rubra', 'Persicae Semen'**	'Astragali Radix'	0.25	0.82	1.71
**'Radix Paeoniae Rubra', 'Carthami Flos'**	'Astragali Radix'	0.29	0.84	1.75

**'Radix Paeoniae Rubra', 'Astragali Radix'**	'Carthami Flos'	0.29	0.84	1.89
**'Carthami Flos', 'Angelicae Sinensis Radix'**	'Astragali Radix'	0.32	0.9	1.87
**'Carthami Flos', 'Astragali Radix'**	'Angelicae Sinensis Radix'	0.32	0.9	1.4
**'Astragali Radix', 'Chuanxiong Rhizoma'**	'Angelicae Sinensis Radix'	0.29	0.89	1.38
**'Astragali Radix', 'Chuanxiong Rhizoma'**	'Carthami Flos'	0.27	0.83	1.87
**'Radix Paeoniae Rubra', 'Persicae Semen'**	'Pheretima'	0.25	0.82	1.49
**'Pheretima', 'Angelicae Sinensis Radix'**	'Persicae Semen'	0.3	0.85	1.64
**'Pheretima', 'Persicae Semen'**	'Angelicae Sinensis Radix'	0.3	0.81	1.26
**'Carthami Flos', 'Astragali Radix'**	'Pheretima'	0.3	0.85	1.54
**'Pheretima', 'Carthami Flos'**	'Astragali Radix'	0.3	0.89	1.86
**'Pheretima', 'Astragali Radix'**	'Carthami Flos'	0.3	0.85	1.9
**'Astragali Radix', 'Chuanxiong Rhizoma'**	'Pheretima'	0.27	0.83	1.51
**'Carthami Flos', 'Persicae Semen'**	'Pheretima'	0.3	0.81	1.46
**'Pheretima', 'Carthami Flos'**	'Persicae Semen'	0.3	0.89	1.73
**'Pheretima', 'Persicae**	'Carthami Flos'	0.3	0.81	1.81

**Semen'**				
**'Pheretima', 'Angelicae Sinensis Radix'**	'Astragali Radix'	0.3	0.85	1.76
**'Pheretima', 'Astragali Radix'**	'Angelicae Sinensis Radix'	0.3	0.85	1.32
**'Persicae Semen', 'Astragali Radix'**	'Pheretima'	0.3	0.85	1.54
**'Pheretima', 'Astragali Radix'**	'Persicae Semen'	0.3	0.85	1.64
**'Pheretima', 'Persicae Semen'**	'Astragali Radix'	0.3	0.81	1.68
**'Pheretima', 'Carthami Flos'**	'Angelicae Sinensis Radix'	0.29	0.84	1.31
**'Radix Paeoniae Rubra', 'Astragali Radix'**	'Pheretima'	0.29	0.84	1.52
**'Pheretima', 'Radix Paeoniae Rubra'**	'Astragali Radix'	0.29	0.84	1.75
**'Radix Paeoniae Rubra', 'Chuanxiong Rhizoma'**	'Pheretima'	0.29	0.84	1.52
**'Pheretima', 'Radix Paeoniae Rubra'**	'Chuanxiong Rhizoma'	0.29	0.84	1.57

Sup Figure S7. shows all the association rules that fulfill the threshold criteria. The first 2 association rules are two-itemset association rules and the following are all three-itemset rules. Three-itemset ssrules refer to the correlation between the occurrence of 2 antecedent herbs and a third consequent herb. This indicates that when the 2 antecedent herbs appear, the subsequent herb is also more likely to co-appear.

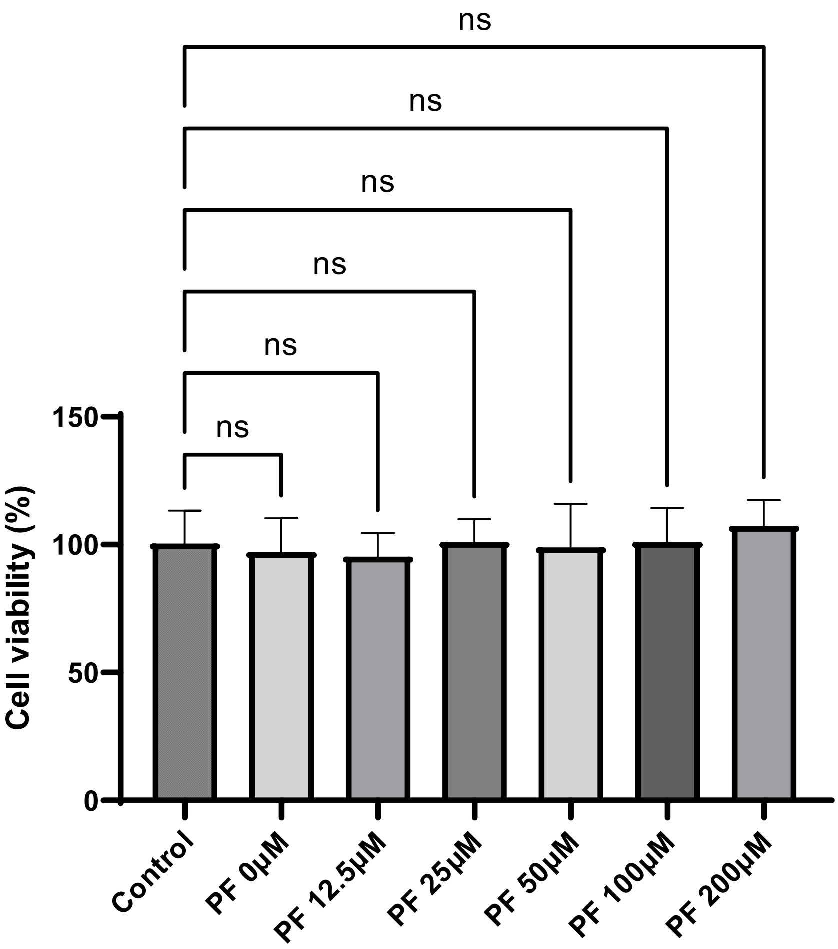

Sup Figure S8. The toxicity evaluation of PF.
